# Le cancer du sein chez l'homme au Maroc: quels impacts psycho-sociaux?

**DOI:** 10.11604/pamj.2013.14.150.2688

**Published:** 2013-04-15

**Authors:** Meryam Ben Ameur El Youbi, Mouna Bourhafour, Hassan Errihani

**Affiliations:** 1 Service d'Oncologie Médicale, Institut National d'Oncologie, Rabat, Maroc

**Keywords:** Cancer du sein, homme, impact psycho-social, Maroc, breast cancer, men, psycho-social impact, Morocco

## Aux éditeurs du Journal Panafricain de Médecine

Le cancer du sein est le cancer le plus fréquent chez la femme à la fois dans les pays développés et les pays en voie de développement. Dans ces derniers, son incidence ne cesse d'accroitre du fait d'une plus longue espérance de vie et d'une meilleure accessibilité aux structures de dépistage et de soins. Cependant, cette maladie typiquement « féminine » peut aussi affecter les sujets de sexe masculin. Le cancer du sein chez l'homme est une affection rare, elle représente environ 1% de tous les cancers du sein et moins de 1% de l'ensemble des néoplasies masculines [1]. Aussi, son incidence a connu une nette recrudescence ces 25 dernières années [2]. Au Maroc, l'incidence du cancer du sein chez l'homme selon nos deux registres nationaux (Registre des cancers de Rabat et Registre des cancers de la région du grand Casablanca) est estimée à 0.8 - 1%. Cette affection encore méconnue du patient marocain revêtit plusieurs aspects psycho-sociaux et culturels intéressants qui méritent d’être investigués. En effet, dans une société arabo-musulmane à l'esprit encore masculin comme la nôtre, la dignité de l'homme peut être grièvement compromise après le diagnostic de cancer du sein, une vraie « honte » pouvant le priver de son statut d'homme viril ou encore le déclasser au rang des femmes.

Dans la littérature, les quelques séries rétrospectives marocaines publiées avaient pour objectif une analyse des caractéristiques cliniques, histologiques et thérapeutiques du cancer du sein chez l'homme [3]. Cependant, aucune étude jusqu’à aujourd'hui ne s'est intéressée à évaluer l'impact psycho-social de cette maladie sur le patient marocain et son entourage. Chez l'homme, le sein n'est qu'un organe vestigial. Une lésion douloureuse ou non se développant dans la région aréolaire sera longtemps ignorée par le patient surtout si celle-ci s'accompagne d'une gynécomastie qu'elle soit unilatérale ou bilatérale. La gynécomastie, l'un des facteurs de risque encore débattu dans la littérature [1], sera plus intimidante pour la personne puisqu'elle peut être visible à autrui et lui conférer l'apparence « d'un homme avec des seins ». Ceci a pour conséquence un retard du diagnostic qui se fera à un stade avancé de l’évolution locale de la maladie. En effet, selon la plus large étude rétrospective menée au Maroc, décrivant 127 cas de cancers de sein chez des patients de sexe masculin, colligés entre 1989 à 2007 à l'Institut national d'oncologie à Rabat au Maroc [4], le délai médian de consultation était de 24 mois. Dans la même étude; 62,5% des patients avaient des tumeurs localement avancées au moment du diagnostic et 29% étaient d'emblée métastatiques. La prise en charge est aussi affectée à plusieurs niveaux; adhésion difficile au plan de soins, mauvaise observance ou interruption du traitement, patients perdus de vu, impactant ainsi négativement les courbes de survie de ces patients qui est nettement inférieure à celle des femmes pour des stades similaires [5].

Le vécu psychique du patient est lourdement affecté, aux troubles psychologiques qui accompagnent habituellement toute annonce du cancer passant par l'acceptation de la maladie jusqu'au deuil; tels que la dépression, l'angoisse et la peur de la mort [6], vont se greffer d'autres troubles : dévalorisation ou perte d'estime de soi, isolement, troubles du schéma corporel et/ou de l'identité sexuelle, pouvant être aussi réprimandant que la maladie en elle-même [7]. Le vécu social du patient qui fuira continuellement le regard des autres est une autre rude épreuve, il va développer un sentiment de rejet ou de non appartenance à la race masculine, phobie sociale et sentiment d'insécurité, il peut aussi adopter une attitude de déni de la maladie, ou encore mentir sur la localisation; « certes il a le cancer, mais pas le cancer du sein ». Le relationnel avec la famille sera aussi très affecté puisqu'il ne se voit plus comme « l'homme de la maison ».

Plusieurs propositions peuvent être faites dans ce sens : tout d'abord, médiatiser le sujet, informer et éduquer la population marocaine concernée. Il faut aussi inciter les hommes à s'auto-palper la région aréolaire en cas d'antécédents familiaux et à consulter en cas d'anomalies. Chez les patients, il est impératif d’évaluer l'impact psycho-social à l'aide de questionnaires de qualité de vie adaptés, pour mieux identifier les problèmes, et répondre aux attentes. On peut aussi proposer de changer la dénomination de la maladie pour une meilleure acceptation de celle-ci. Finalement, on n'insistera jamais assez sur l'importance de la prise en charge psychologique du patient et de son entourage, qui doit faire partie du traitement et être multidisciplinaire faisant intervenir plusieurs acteurs (médecins, infirmiers, psychologues et assistantes sociales).

## Conclusion

La plupart des séries publiées dans la littérature décrivent le cancer du sein chez l'homme comme étant une maladie agressive avec un pronostic réservé, sans pour autant intégrer l'ensemble de ces paramètres psychologiques et culturels au sein d'un contexte social particulier. Des efforts d′éducation et d′information de la population marocaine doivent être conjugués à une prise en charge psychologique adéquate du patient et de sa famille afin d'améliorer le vécu et le pronostic du patient marocain atteint de cancer du sein.
